# Modifications to established fiber methods may be required to quantify cellulose from flow aids in grated Parmesan cheese

**DOI:** 10.3168/jdsc.2020-18275

**Published:** 2020-09-02

**Authors:** C.E. Tyl, L. Vazquez Portalatin, T.C. Schoenfuss

**Affiliations:** Department of Food Science and Nutrition, University of Minnesota, Saint Paul 55108

## Abstract

Reliable detection and quantification of flow-aid concentrations in grated cheese is warranted for both quality assurance and to prevent fraud, yet no official method exists. This study evaluated enzymatic–gravimetric methods that quantify insoluble dietary fiber, as well as near-infrared spectroscopy (NIR), for their suitability to measure cellulose and flow-aid concentrations in ground Parmesan with known amounts of commercial flow-aid preparations. The range of flow-aid concentrations spanned 0 to 5.01 g/100 g of cheese, corresponding to 0 to 1.39 g/100 g of cellulose. Use of the total dietary fiber assay, with or without modifications, consistently overestimated flow-aid concentrations by formation of aggregates that were presumably difficult to digest. Increasing protease amounts reduced but did not eliminate this issue. In contrast, the integrated dietary fiber assay and NIR spectroscopy were suitable methods for quantification of cellulose and flow aid. However, analyzing control cheeses without flow aid proved difficult with both methods. For these samples, the integrated dietary fiber assay and NIR spectroscopy calibrated for cellulose gave higher than actual values with poor precision (0.50 ± 0.36 and 0.38 ± 0.22 g/100 g, respectively). However, NIR calibrations for flow aid (which also contained starch), instead of cellulose, specifically yielded more accurate results for samples with 0 g/100 g of flow aid (0.06 ± 0.14 g/100 g). In general, prediction of higher concentrations of flow aid via NIR spectroscopy had lower accuracy than with the integrated dietary fiber assay; however, results obtained via NIR spectroscopy had lower variability. The total dietary fiber assay always overestimated cellulose in ground Parmesan, and further modifications are necessary to obtain accurate results. Differences between the pH at which protein digestion occurs (lower for the integrated dietary fiber assay than the total dietary fiber assay) may have contributed to differences in results.

## Highlights

• Flow aids containing cellulose are used to prevent caking in hard-grated cheeses.
• Quantification of cellulose in parmesan by AOAC 991.43 overestimated the amount.
• Quantification of cellulose in parmesan by AOAC 2011.25 was accurate.
• The methods vary in pH and acid type during protein solubilization.
• Fourier transform-near infrared calibrated for flow aid in grated parmesan accurately predicted cellulose.

## Graphical Abstract

**Figure:**
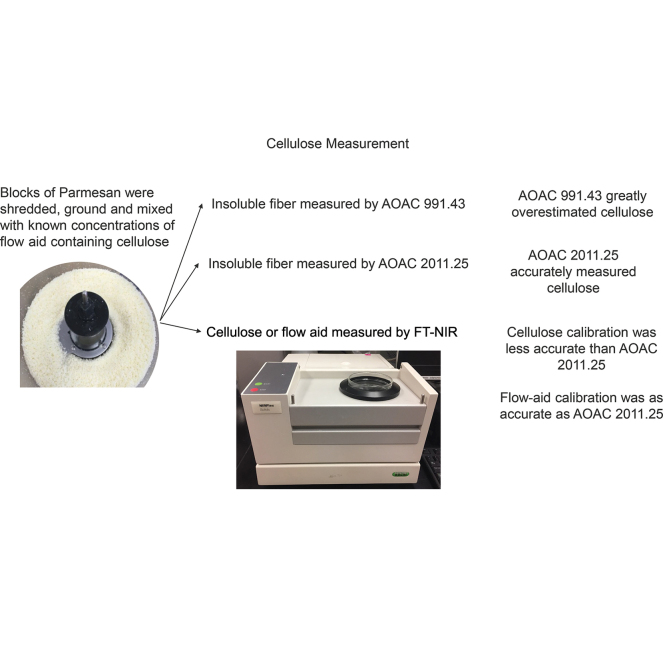
**Summary:** Reliable detection and quantification of flow-aid concentrations in grated cheese is warranted for both quality assurance and to prevent fraud, but no official method exists. Although the analysis time for AOAC method 2011.25 is long, the method is capable of quantifying flow-aid concentrations from 1.96 to 5.01 g/100 g, and thus is suitable for commercial samples.

Flow aids added to grated cheese usually contain cellulose, starch, or a combination thereof, and are primarily added to prevent clumping of cheese in the package or as a vehicle for antimycotics and oxygen scavengers to maintain cheese quality (Ture et al., 2011; Singh et al., 2015a,b). The maximum concentration of flow aid is not regulated by US law; however, for shredded cheddar and mozzarella cheese to meet USDA standards, 2% based on weight is the maximum allowed (USDA, 2001, 2018). The lack of an official analytical method for accurate flow-aid quantification in cheese can make it difficult for cheese manufacturers to adhere to these limits. In addition, it impairs monitoring of the levels by regulatory agencies, because no studies have been done to validate the accuracy of AOAC International-approved fiber methods to measure cellulose in cheese.

Near-infrared (**NIR**) spectroscopy is a technique routinely used in analytical laboratories and has successfully been used to analyze the composition of dairy products (ISO/IDF, 2006) and adulterants (Poonia et al., 2017). Usually, NIR spectroscopic analysis is combined with chemometric methods. Near-infrared spectroscopy has the advantage of being a simple, rapid, and nondestructive method that requires little preparation (Subramanian and Rodriguez-Saona, 2009). However, it does require extensive calibration with a large sample set and can be sensitive to variability caused by differences in composition and sample presentation (e.g., differences in particle size, inclusion of air bubbles, temperature of scanning; Williams, 2008).

Cellulose quantities in shredded cheese can also be determined gravimetrically after digestion and separation from other cheese constituents. This can be achieved via official dietary fiber methods, which use proteases in combination with α-amylases and amyloglucosidases (AOAC International, 2005, 2012). Insoluble and soluble residues can be differentiated (depending on the point of ethanol addition to precipitate high-molecular-weight soluble fibers) and quantified gravimetrically. Because cellulose is practically insoluble in water, due to formation of intra- and intermolecular hydrogen bonds and resulting alignment into fibrils, it is present in the insoluble dietary fiber (**IDF**) portion (Capuano, 2017).

The aim of this study was to assess whether NIR spectroscopy and enzymatic–gravimetric analysis via official dietary fiber methods (with slight modifications) allowed for quantifying cellulose-containing flow-aid concentrations in shredded Parmesan. Previously, we successfully measured cellulose in cheddar and mozzarella by AOAC method 991.43 (AOAC International, 2005) and achieved accurate results (Zumbusch, 2017). However, we had issues with measuring cellulose in Parmesan. We undertook this study to determine whether this method could be modified to work or whether a different fiber method would be more accurate. Measurements were performed on Parmesan samples containing 0 to 5.01% flow aid (Free Flow 70, Allied Blending LP, Keokuk, IA), corresponding to 0 to 1.39% cellulose. This range was based on 2% flow aid being the maximum allowed under USDA requirements for shredded mozzarella (USDA, 2018) and the expectation that twice this concentration could be encountered. The flow aid also contained 40.23% starch. Parmesan blocks were obtained from local grocery and food-service stores and shredded in a food processor using the shredding disc. The shredded cheese was then divided into bags of 200-g samples. Flow aid was added, mixed by shaking the bag by hand, and then blended in a food processor for 1 min. Cheese was stored refrigerated. For NIR sample presentation, ground prepared cheese was brought to room temperature before being formed into balls of 60 g, which were put into the center of a glass Petri dish (15 × 100 mm). A circular disc (9.5-cm diameter) of high-density polyethylene was used as a press plate. A 6.35-cm C-clamp was clamped around the press plate and the bottom of a force plate (FP-BTA, Vernier Software and Technology, Beaverton, OR) and was pressed for 1 min at a force of 250 to 280 N. For each flow-aid concentration, 3 replicates were analyzed. Near-infrared spectroscopic calibration via partial least squares was done by analyzing 702 samples of Parmesan on a NIRFlex Solids spectrometer (Buchi, New Castle, DE) using NIRCal 5.2 Chemometric software (Buchi). Spectra were divided into calibration and validation sets (Figure 1). These sets were composed of a continuous pattern of 6 spectra for calibration and 3 spectra for validation. The wavelengths from 8,900 to 10,000 cm^−1^ were eliminated, a standard normal variate pretreatment was performed, and 15 principal components were used.

**Figure 1:**
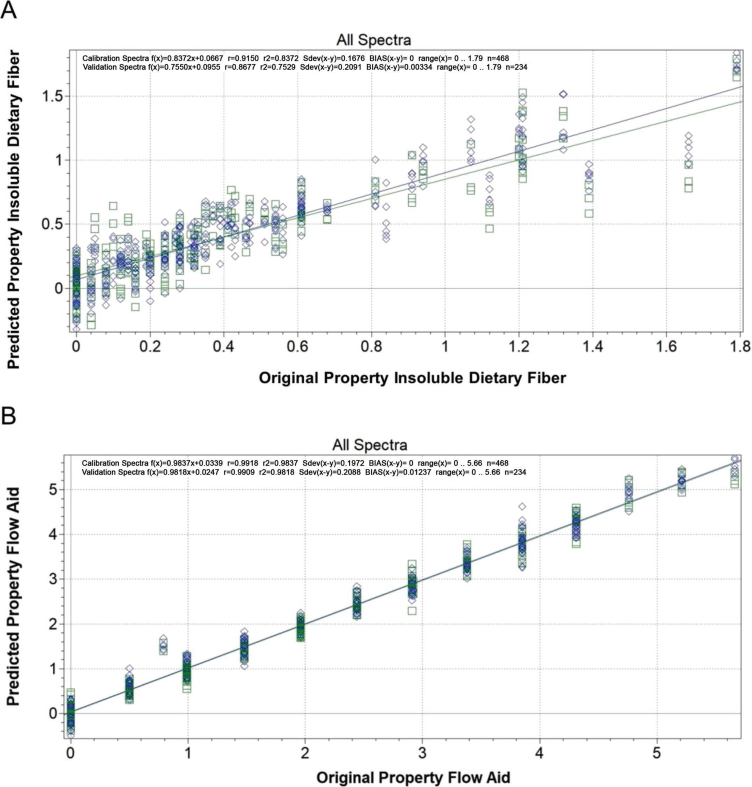
Calibration curves for near-infrared spectroscopic analysis of grated Parmesan cheese for quantifying (A) cellulose as insoluble dietary fiber and (B) cellulose-containing flow aid. Blue diamonds indicate calibration spectra, and green squares indicate validation spectra.

Enzymatic–gravimetric quantification of cellulose was performed by obtaining IDF via AOAC method 991.43 (AOAC International, 2005) and AOAC method 2011.25 (AOAC International, 2012) with slight modifications. As recommended by AOAC method 991.43, a defatting step was included before enzymatic digestion, where cheese (3 g) was sonicated with 40 mL of hexane for 5 min, centrifuged at 2,060 × *g* for 5 min, and the upper organic phase discarded. The procedure was repeated twice and followed by overnight air-drying after the third extraction step. Yields for defatted cheese were in the range of 44 to 46%. The amounts of α-amylase (50 μL) and amyloglucosidase (200 μL) used for digestion via AOAC methods 991.43 and 2011.25 were the same as specified by the methods for sample sizes of 1 g. Four dietary fiber analysis methods were compared. Method A was in accordance to AOAC method 991.43 for sample sizes of 1 g. Method B was the same as method A, except the amount of protease was increased to 200 μL to ensure complete protein digestion, and the digestion time was increased from 30 min to 1 h. Method C involved switching the order of protein and starch digestion, but otherwise followed AOAC method 991.43. Method D corresponded to AOAC method 2011.25 for a sample size of 1 g, but used 300 μL of protease instead of 100 μL and a protein digestion time of 1 h instead of 30 min. Samples were analyzed at least in duplicate and corrected for residual protein and ash as specified by the official methods, with the exception that for protein correction (via the Kjeldahl method), a protein conversion factor of 6.38 instead of 6.25 was used, as this value is more commonly representative for dairy proteins (Jones, 1941).

The IDF values were consistently overestimated by AOAC methods A, B, and C based on 991.43, even when higher amounts of protease and longer incubation periods were employed (Table 1). Although it could be argued that protease additions need to be tripled based on the original sample size of 3 g of cheese (as opposed to 1 g of sample required by the method), even in additional experiments involving a 3-fold increase in protease, the formation of aggregated particles that collected in the crucible were not completely eliminated. When a cheese sample containing 4.76 g of flow aid/100 g of cheese was analyzed via AOAC method 991.43, but with 3× the amount of protease and 1 h of digestion, IDF contents indicated a flow-aid concentration of 11 g/100 g of cheese (results not shown). The reason for starting with 3 g instead of 1 g of sample is related to the low IDF amounts in low flow-aid concentrations (approximately 15–40 mg in our sample set); their mass is obtained by difference (i.e., the weight of the crucible plus filter aid Celite before and after filtration and drying). When analyzing the insoluble residues for protein via the Kjeldahl method (correcting for proteins is part of the method), much of the precipitate could be accounted for. However, substantial amounts of presumed dietary fiber remained that far surpassed the amounts of cellulose provided by the flow aid.

**Table 1:** Insoluble fiber concentrations in ground Parmesan calculated either gravimetrically or via near-infrared (NIR) spectroscopy after calibrating for cellulose^1^

Insoluble fiber (g/100 g of cheese)	Method A^2^	Method B^3^	Method C^4^	Method D^5^	NIR
0	3.04^a^ ± 0.61	0.93^b^ ± 0.11	2.12^a^ ± 0.33	0.14^c^ ± 0.10	0.38^c^ ± 0.22
0.544	2.99^a^ ± 0.18	3.11^a^ ± 1.00	ND	0.52^b^ ± 0.03	0.76^b^ ± 0.17
0.68	ND	1.24^b^ ± 0.33	2.02^a^ ± 0.43	0.67^b^ ± 0.35	0.62^b^ ± 0.06
0.807	6.77^a^ ± 0.40	3.41^b^ ± 0.34	ND	0.83^c^ ± 0.02	1.10^c^ ± 0.07
1.39	ND	2.12^a^ ± 0.26	2.30^a^ ± 0.21	1.35^b^ ± 0.04	1.21^b^ ± 0.05

a–c Different superscripts signify differences across rows according to LSD test (*P* < 0.05).

1 Gravimetric values are based on mass of insoluble residues after enzymatic digestion according to official dietary fiber methods performed with slight modifications as specified; all values are reported as means ± SD (g/100 g of cheese, wet basis) of at least duplicate determinations; NIR spectroscopic determinations were based on calibrations with 15 principal components; ND = not determined due to insufficient sample amounts.

2 Determined using method 991.43, AOAC International (2005).

3 Determined using method 991.43 (AOAC International, 2005), but with the amount of protease increased from 100 to 200 μL.

4 Determined using method 991.43 (AOAC International, 2005), but switching the order of protein and starch digestion.

5 Determined using method 2011.25, AOAC International (2012), for a sample size of 1 g, but using 300 μL of protease instead of 100 μL and a protein digestion time of 1 h instead of 30 min.

In contrast, method D based on AOAC method 2011.25 was well-suited to quantify the insoluble residues (Table 1), which contained very little residual protein. Both methods include a heating step in a boiling water bath; for AOAC method 991.43, it is used to digest starch with a thermostable α-amylase, whereas in AOAC method 2011.25, it is used to inactivate α-amylase and amyloglucosidase (i.e., the starch digestion has already been completed). In both methods, the protein digestion occurs right after these heating steps, after samples have equilibrated to 60°C. The formation of cheese aggregates was observed during the heating step in AOAC method 991.43, but not for AOAC method 2011.25. This difference might be due to the fact that the pH is different during this treatment. AOAC method 991.43 uses a 0.05 *M* 2-(*N*-morpholino)ethanesulfonic acid/tris(hydroxymethyl)aminomethane buffer at pH 8.2, whereas AOAC method 2011.25 uses a 0.05 *M* maleic acid buffer at pH 6. Although the molarity is the same, maleic acid contains 2 carboxyl groups, and thus the ionic strength (i.e., 1/2 Σ *c_i_z_i_*^2^, with *c* being concentration and *z* being charge) is higher. Additionally, the maleic acid buffer contains 2 m*M* calcium chloride. Generally, calcium will enhance interactions between caseins. Protein solubility of caseins is typically higher at pH 8.2 than pH 6, though it is dependent on casein type and, to a lesser degree, on temperature (Post et al., 2012). However, calcium solubility is increased at lower pH, allowing colloidal calcium phosphate to leave the casein micelle. In the presence of citrate, higher concentrations of calcium ions are reported in the aqueous than the micellar phase below pH ∼6.75 (Gaucheron, 2011). Parmesan cheese has a very high concentration of calcium (1,184 mg/100 g) compared with cheeses such as mozzarella (697 mg/100 g) and cheddar (710 mg/100 g; USDA, 2019). The lower pH combined with the higher ionic strength are likely to have caused the solubilization of calcium and inorganic phosphate and allowed for the removal of aggregates from the insoluble fraction in AOAC method 2011.25 when compared with AOAC method 991.43.

Although NIR spectroscopy measurement using our developed calibrations for flow aid and cellulose did not overestimate IDF, its results did not match the accuracy of AOAC method 2011.25 for samples containing 2.91 and 5.01 g/100 g cheese (Table 1). Two calibration approaches were used: 1 for cellulose and 1 for total amount of flow aid. The calibration for cellulose had an R^2^ of 0.8372, and that for flow aid was 0.9837. It can be seen in Figure 1 that the flow-aid calibration and validation values are much closer to their predicted values than in the cellulose (IDF) calibration. As a result, the flow-aid calibration gave results that matched flow-aid concentrations well for samples with 1.96 and 2.44 g/100 g of flow aid, and the standard deviations were much lower than for the enzymatic–gravimetric procedures (Table 2). More samples with higher cellulose concentrations would need to be added to the cellulose-only calibration to make conclusions about its ability to quantify it at higher concentrations. However, NIR calibrations for cellulose, as well as all enzymatic–gravimetric methods, overestimated the flow-aid concentrations for control cheese samples without flow aid. For this reason, more replicates were analyzed via methods A, B, and D; however, standard deviations remained large and accuracy low. Thus, the data indicate that for no to low concentrations of flow aid, NIR spectroscopy is better suited once calibration has been optimized. Although the analysis time for AOAC method 2011.25 is long, it is capable of quantifying flow-aid concentrations from 1.96 to 5.01 g/100 g, corresponding to commercial samples. More work is needed to establish limits of detection and quantification, as well as working ranges, on Parmesan and other cheeses. Further study to understand what is causing the precipitation and overestimation of IDF for AOAC method 991.43, and possible corrections to the method, is warranted as it is the method most commonly used to measure cellulose in food.

**Table 2:** Flow-aid concentrations in ground Parmesan calculated either gravimetrically or via near-infrared (NIR) spectroscopy^1^

Flow aid (g/100 g of cheese)	Method A^2^	Method B^3^	Method C^4^	Method D^5^	NIR
0	10.97^a^ ± 2.19	3.36^c^ ± 0.40	7.65^b^ ± 1.20	0.50^d^ ± 0.36	0.06^d^ ± 0.14
1.96	10.77^a^ ± 0.66	11.21^a^ ± 3.60	ND	1.86^b^ ± 0.12	2.03^b^ ± 0.10
2.44	ND	4.48^b^ ± 1.18	7.27^a^ ± 1.55	2.42^b^ ± 1.27	2.45^b^ ± 0.07
2.91	24.42^a^ ± 1.46	12.31^b^ ± 1.21	ND	2.99^c^ ± 0.07	2.67^c^ ± 0.02
5.01	ND	7.66^a^ ± 0.95	8.30^a^ ± 0.75	4.87^b^ ± 0.15	4.14^b^ ± 0.07

a–d Different superscripts denote differences among means across rows according to LSD test (*P* < 0.05).

1 Concentrations for flow aid were calculated by multiplying insoluble fiber values from Table 1 by the factor corresponding to the cellulose concentration of flow aid, or by calibrating the NIR with flow aid; all values are reported as means ± SD (g/100 g of cheese, wet basis) of at least duplicate determinations; NIR spectroscopic determinations were based on calibrations with 15 principal components; ND = not determined due to insufficient sample amounts.

2 Determined using method 991.43, AOAC International (2005).

3 Determined using method 991.43 (AOAC International, 2005), but with the amount of protease increased from 100 to 200 μL.

4 Determined using method 991.43 (AOAC International, 2005), but switching the order of protein and starch digestion.

5 Determined using method 2011.25, AOAC International (2012), for a sample size of 1 g, but using 300 μL of protease instead of 100 μL and a protein digestion time of 1 h instead of 30 min.
